# A Critical Review of Alpha Radionuclide Therapy—How to Deal with Recoiling Daughters?

**DOI:** 10.3390/ph8020321

**Published:** 2015-06-10

**Authors:** Robin M. de Kruijff, Hubert T. Wolterbeek, Antonia G. Denkova

**Affiliations:** Radiation Science and Technology, Delft University of Technology, Mekelweg 15, 2629 JB Delft, The Netherlands; E-Mails: r.m.dekruijff@tudelft.nl (R.M.K.); h.t.wolterbeek@tudelft.nl (H.T.W.)

**Keywords:** alpha-emitters, recoils, radionuclide therapy

## Abstract

This review presents an overview of the successes and challenges currently faced in alpha radionuclide therapy. Alpha particles have an advantage in killing tumour cells as compared to beta or gamma radiation due to their short penetration depth and high linear energy transfer (LET). Touching briefly on the clinical successes of radionuclides emitting only one alpha particle, the main focus of this article lies on those alpha-emitting radionuclides with multiple alpha-emitting daughters in their decay chain. While having the advantage of longer half-lives, the recoiled daughters of radionuclides like ^224^Ra (radium), ^223^Ra, and ^225^Ac (actinium) can do significant damage to healthy tissue when not retained at the tumour site. Three different approaches to deal with this problem are discussed: encapsulation in a nano-carrier, fast uptake of the alpha emitting radionuclides in tumour cells, and local administration. Each approach has been shown to have its advantages and disadvantages, but when larger activities need to be used clinically, nano-carriers appear to be the most promising solution for reducing toxic effects, provided there is no accumulation in healthy tissue.

## 1. Alpha Emitting Radionuclides in the Clinic

Alpha-emitting radionuclides are a very promising type of radiotherapeutic agents showing great potential in the treatment of a broad range of malignancies [1,2,3,4,5,6,7,8,9,10,11]. Due to the short penetration depth of alpha particles, they are capable of destroying tumours while causing very limited damage to the surrounding healthy tissue. In fact, an emitted alpha particle will not travel much further than about 6 cell diameters (*i.e.*, 50–100 µm). On the other hand, their high linear energy transfer (LET) gives them an increased relative biological effectiveness (RBE) [12] as compared to other radionuclide therapies. Just a few alpha particles through a cell nucleus are sufficient to destroy a cell, as cell death due to alpha-radiation is largely independent of oxygenation or active cell proliferation [13]. Furthermore, when alpha-emitting radionuclides are targeted to specific tumour cells in the body, they can be very effective in destroying metastases, which are difficult to treat by currently employed techniques.

Alpha radionuclide therapy has recently been introduced in the clinic, where it has seen a number of successes. Several clinical trials have been done with ^213^Bi (bismuth), ^221^At (astatine) and ^212^Pb (lead) [1], and pre-clinical studies are being carried out with ^149^Tb (terbium) [14]. Using tumour-specific monoclonal antibodies, ^213^Bi has shown promise in the treatment of metastatic melanoma [2,3] and produced remissions in patients with acute myeloid leukemia [4,5,6]. A phase I study was completed on the use of locally injected ^213^Bi-DOTA-substance P in the treatment of small gliomas. The patients received 1.07–2.00 GBq in one cycle or a total of 7.36 GBq in four cycles of ^213^Bi-DOTA-substance P, of which more than 96% was retained at the tumour site. Most benefit has been seen in relatively small tumours, as sufficient intratumoural distribution has been achieved causing the tumours to be completely necrotic demarcated making it possible for non-operable gliomas to be surgically removed [7]. ^221^At is also proving to be a promising alpha-emitter. A pilot study amongst patients with recurrent malignant brain tumours has shown the feasibility of the administration of ^211^At-ch81C6 into the surgically created resection cavity of patients, and has demonstrated minimal toxicity [8]. Women with recurrent ovarian carcinomas have been treated with varying therapeutic doses (33.6–119.2 MBq) of ^211^At bound to antibody fragments without detectable adverse effects, indicating that therapeutic doses can be delivered to micrometastases by the intraperitoneal administration of the ^211^At-MX35 F(ab’)_2_ [9].

Despite of the clear promise of alpha radionuclide therapy, its use is limited to easily accessible tumours due the short half-lives of the applied isotopes: from 45.6 min for ^213^Bi up to 7.2 h for ^211^At. Less easily accessible sites, where the targeting agent is taken up slowly, or solid tumours where a longer penetration time of the radiopharmaceutical is necessary, will need longer-lived alpha emitting radionuclides for optimal treatment [15]. So far, three long-lived alpha-emitters, namely ^224^Ra, ^223^Ra, and ^225^Ac, have made their appearance in the clinic. One of the first therapeutic uses of alpha-emitting radionuclides has been the intravenous injection of ^224^Ra-chloride in ankylosing spondylitis (AS) patients (a chronic inflammatory rheumatic disease). Between 1948 and 1975, 1588 patients were treated with repeated injections, receiving an activity of approximately 50 MBq. Amongst these patients, the cause of death could be determined for 1006. Although at the time positive therapeutic effects were confirmed for the AS patients, many patients, especially those aged below 21 when treated, developed malignant bone tumours. This increase in myeloid leukaemia as compared to the control group could be explained by the deposition of ^224^Ra into the bone. An increase in kidney and thyroid cancer was also observed, as compared to a control group [16,17]. In Germany, the pure ^224^Ra- chloride compound was approved again for the intravenous administration in AS patients in 2000, under the name ^224^Spondyl*AT*^®^ [18]. The dose was much lower than what was used before, as patients now received total activities of only 10 MBq, with 1 MBq per injection [12]. Though the ^224^Ra treatment seemed to reduce medical costs and lost productivity [19], they could not deliver the clinical evaluation on time so it was discontinued in 2005 [20,21]. Since then ^224^Ra has not been used in clinical settings.

A comprehensive review has recently been published by Jadvar and Quinn [11] on the clinical trials of ^223^Ra dichloride (Xofigo^®^, formerly Alpharadin^®^) in the treatment of bone metastasis following castration-resistant prostate cancer. A short summary will be provided here for completeness. From 2008 to 2011 a total of 921 patients from 19 countries participated in a double-blind randomized phase 3 trial (ALSYMPCA). The patients in the treatment arm received six injections of 50 kBq/kg ^223^Ra, the patients in the placebo arm six injections with saline. A 3.6-month prolonged survival as compared to the placebo group has been observed [22]. On the completion of the trial, the treatment became FDA-approved. Dosimetric considerations following the intravenous injection of ^223^Ra dichloride report a dose of 16 Gy to the bone endosteum, and 1.5 Gy to the red bone marrow, while other organs received a lower dose [23]. Biodistribution experiments have shown that less than 2% of the daughter nuclides migrate away from the bone surface [24] Currently, Phase II trials are on-going for the use of ^223^Ra dichloride to help control breast cancer that has spread to the bones of breast cancer patients [25,26], although to date no results of these studies have been published.

The third radionuclide with alpha-emitting daughters that made it to the clinic is ^225^Ac. ^225^Ac-Labeled Humanized Anti-CD33 Monoclonal Antibody HuM195 (Actimab-A) has been used to treat patients with advanced myeloid malignancies. A Phase I trial has demonstrated that it is safe to use at doses ≤ 0.1 MBq/kg. Although dose-limiting toxicity (DLT) has occurred in one patient receiving 0.1 MBq/kg and two patients receiving 0.15 MBq/kg [27], blast reductions of more than 50% have been observed in 6 out of 12 patients [28]. No acute toxicities have been seen other than transient grade 2/3 liver function abnormalities, and no apparent damage to kidneys has been observed [10]. In a subsequent Phase-I trial, elderly patients with a total administered activity between 2.5 and 7.4 MBq have been treated. Bone marrow blast reductions have been seen in four out of six patients, but unfortunately no complete remissions have been reported. DLT has occurred in one patient with grade-4 thrombocytopenia, and other toxicities included grade 3 febrile neutropenia (n = 5) and pneumonia (n = 1). The FDA has cleared the compound for subsequent Phase I/II trials, where response rate, progression-free survival and overall survival will be assessed [29].

**Figure 1:**
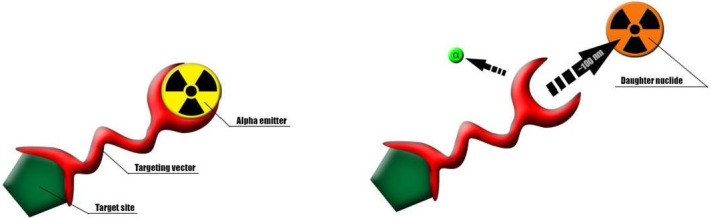
Schematic representation of a recoiling daughter radionuclide detaching from a targeting agent as a consequence of alpha decay.

There is potentially a future for radionuclides with multiple alpha-emitting daughters like ^225^Ac (t_1/2_ = 10.0 days) or ^223^Ra (t_1/2_ = 11.4 days). However, toxicity is clearly still an issue due to the recoil energy the daughters experience upon alpha decay. This energy is in most cases at least 100 keV, more than 1000 times larger than the binding energy of any chemical compound. This simply means that bond rupture will always occur subsequent to alpha decay, implying that the released daughters, which are often themselves alpha emitters, might cause considerable harm (as seen in some of the clinical trials with e.g. AS patients [17]) since it will no longer be bound to a targeting moiety (Figure 1). Considering the presence of different proteins in blood and their high binding capacity the chance that the radionuclide will re-associate back with its original ligand is rather small. Therefore, in this review we will discuss the different alpha-emitting radionuclides and the strategies to limit their toxicity.

## 2. *In Vivo* Alpha Generator Radionuclides and Their Decay Chains

In Table 1 we have listed the potential alpha-emitting radionuclides having successive alpha-emitting daughters together with their decay characteristics and the recoil energy that the daughter radionuclides receive after decay. In several decay chains, multiple radionuclides can be used as *in vivo* generators for alpha-radionuclide therapy. These radionuclides are presented in bold-face in Table 1.

**Table 1:** Alpha radionuclides that can be used as *in vivo* generators and their decay characteristics [30].

Radionuclides and Their Decay Chain	Half-Life	Decay	E_α_ (MeV) *	Calculated E_R_ (keV) *
^225^**Ac**	9.9 days **	α(100%)	5.8	-
^221^Fr	4.9 min	α(100%)	6.3	105.5
^217^At	32 ms	α(99.98%)/β^−^(0.01%)	7.1	116.9
^213^Bi	45.6 min	α(2%)/β^−^(98%)	-	132.8
^213^Po	4.2 µs	α(100%)	8.4	-
^209^Pb	3.3 h	β^−^ (100%)	-	160.4
^209^Bi	stable	-	-	-
^227^**Th**	18.7 days	α(100%)	6	-
^223^**Ra**	11.4 days	α(100%)	5.7	108.4
^219^Rn	4.0 s	α(100%)	6.8	104.5
^215^Po	1.8 ms	α(100%)	7.4	126.9
^211^Bi	2.2 min	α(99.7%)/β^−^(0.3%)	6.6	140.1
^207^Tl	4.8 min	β^−^ (100%)	-	128.1
^207^Pb	stable		-	-
^228^**Th**	1.9 years	α(100%)	5.4	-
^224^**Ra**	3.7 days	α(100%)	5.7	96.9
^220^Rn	55.6 s	α(100%)	6.3	103.4
^216^Po	0.15 s	α(100%)	6.8	116.5
^212^Pb	10.6 h	β^−^(100%)	-	128
^212^Bi	60.6 min	α(36%)/β^−^(64%)	6.1/-	-
^208^Tl/^212^Po	3.0 min/0.3 µs	β^−^(100%)/α(100%)	-/8.8	116.5/-
^208^Pb	stable	-	-	-
^230^**U**	20.8 days	α(100%)	5.9	-
^226^**Th**	31 min	α(100%)	6.3	104.3
^222^Ra	38 s	α(100%)	6.5	114.2
^218^Rn	35 ms	α(100%)	7.1	120.4
^214^Po	164 µs	α(100%)	7.7	133.4
^210^Pb	22.3 years	β^−^(100%)		146.5
^210^Bi	5.0 days	β^−^(100%)		-
^210^Po	138.4 days	α(100%)	5.3	-
^206^Pb	stable			-

* The alpha and recoil energies have been rounded up;** this value is from Pommé *et al*. [31].

^228^Th (thorium) and ^224^Ra are the products of two decay chains: The one of ^232^Th (^232^*Th* $\overset{\alpha }{\to }$^228^*Ra* $\overset{\beta -}{\to }$^228^*Ac* $\overset{\beta -}{\to }$^228^*Th*) and the decay of ^232^U (uranium) (^232^*U* $\overset{\alpha }{\to }$^228^*Th*). Starting from ^232^Th, ^228^Ra is extracted and left to decay to ^228^Th, which is then applied to prepare a ^224^Ra generator. ^228^Th can also be obtained by successive neutron capture of ^226^Ra, resulting in ^228^Ra which decays in two beta decay steps to ^228^Th. ^228^Th, due to its long half-life, is only used to generate ^224^Ra. This decay series produces four alpha particles, counted from ^224^Ra, and four recoiling daughters of which ^22^^0^Rn, ^212^Pb and ^2^^08^Tl (thallium) are the most important ones in terms of half-life. In addition to the recoils released by the alpha decay, ^212^Bi, which is also an alpha emitter, can be freed due to the internal conversion of γ-rays emitted by the excited ^212^Bi nuclides. Thus, in this decay series there are actually five daughters that might be harmful [32].

^227^Th and ^223^Ra are both decay products of ^227^Ac, which is extracted from the mill waste of ^235^U mines. ^227^Th can be separated from its mother using anion exchange chromatography [33], while ^223^Ra is typically supplied from a ^227^Ac generator containing an actinide chromatographic resin (Dipex 2) [34]. This decay chain generates five alpha particles and recoiling daughters of which ^223^Ra, ^211^Pb, and ^2^^07^Tl, are the most essential. Radium is in this case the most hazardous one due to its considerably long half-life and the fact that it is part of a decay chain having four alpha particles. This is also the reason to directly use ^223^Ra for treatment rather than ^227^Th, although it comes with the additional challenge of finding a chelate able to make a stable complex with radium. In addition, the increased demand for ^223^Ra will require the development of alternative production routes of ^227^Th.

^225^Ac and ^213^Bi are typically extracted from ^229^Th, originating from ^233^U, produced by neutron capture of ^232^Th (^233^*Th* $\overset{\beta -}{\to }$^233^*Pa* $\overset{\beta -}{\to }$^233^*U* $\overset{\alpha }{\to }$^229^*Th*). Due to increasing demand of ^225^Ac, shortage via this production route is expected and alternative methods are being investigated, from which the proton irradiation of ^226^Ra targets appears to be the most promising process [35]. An alternative to this route is the bombardment of ^232^Th targets by protons having energy of less than 200 MeV, as researched by Weidner *et al.* [36]. The ^225^Ac chain has four major alpha particles and recoiling daughters, from which ^221^Fr, ^213^Bi and ^209^Pb are long-lived enough to induce undesired damage due to subsequent alpha or beta emission.

^23^^0^U is produced in cyclotrons typically by the proton bombardment of ^232^Th, resulting in ^23^^0^Pa which decays to ^23^^0^U, allowing the extraction of the carrier-free product [35]. The ^23^^0^U chain has five alpha particles before reaching the long-lived beta emitter ^21^^0^Pb. There are in this case only two recoil daughters of concern^226^Th and ^222^Ra, if we do not count ^21^^0^Pb, a. An additional problem here, similar to radium, is the availability of appropriate chelates allowing *in vivo* applications with little loss of uranium.

## 3. Distribution of Recoil Daughters in the Body

There are three processes that contribute to the distribution of the recoiling daughters: (1) distance covered due to recoil energy, (2) diffusion processes and (3) active transport such as convection or blood flow. The importance of each process will depend on the location where the recoil atoms are released. The instantaneous energy that a recoil atom gets will make it cover on average around 100 nm and is therefore mostly responsible for the breaking of chemical bonds or the possible escape from carriers [37]. It is the indirect process that allows the recoils to acquire different speciation and distribution than originally intended. Diffusion transport becomes important once the radionuclide reaches certain tissues or organs, such as tumours or kidneys for instance, and will be dependent on the medium. It will thus differ between diffusion in blood-like medium and in the extra/intracellular matrix, as it would be subject to the interaction of the ions with blood or cell components. Most of the time the recoils will be released in the blood stream and their eventual fate will be determined by their affinity for certain organs/tissues provided that they live long enough to reach their biological destination. Table 2 shows a summary of the target organs of several of the elements associated with recoiling daughters.

**Table 2:** Major targeted organs based on 24 h distribution after intravenous injection (IV).

Element	Major Targeted Organs *
Francium	primarily kidneys [38]
Bismuth	30% urine, 40% kidney, 30% other organs [39]
Radium	25% bone surface, 45% soft tissue, 30% excreted via large intestine [12,24]
Radon	soft tissue to blood: 100 day^−1^, exhaled 1 min^−1^, bone to blood: 0.36 d^−1^ [40]
Lead	55% blood, 15% liver, 10%–15% skeleton, kidneys 4% after 1 day [40]
Polonium	28% liver, 28% kidneys, 10% red bone marrow, 5% spleen [41]

Several studies have been dedicated for establishing the fate of alpha emitters, including mother and daughter ions. For instance, the radiotoxicity of ^225^Ac has been evaluated in mice. Biodistribution studies have been done with ^225^Ac chelated to four different compounds: cyclohexyl diethylenetriamine pentaacetic acid (CHX-DTPA), 1,4,7,10,13-pentaazacyclopentadecane-N,N′,N′′,N′′′,N′′′′-pentaacetic acid (PEPA), ethylene diamine tetraacetic acid **(**EDTA) and acetate. In this work it has been found that ^225^Ac mainly goes to the liver, but also accumulates in the femur, kidneys and heart, and although its concentration in the liver and bone increases with time, it is cleared from the kidney and the heart. The ^225^Ac-acetate complex has had a 5.5 times higher %ID/g in the liver than the ^225^Ac-CHX-DTPA and the ^225^Ac-PEPA complexes. It must be noted that the biodistribution of ^225^Ac has been determined immediately after sacrifice using the 218 keV gamma emitted by ^221^Fr, although with its 4 min half-life ^221^Fr could have recoiled and diffused away from the location of the mother isotope [42]. A different study by G.J. Beyer *et al.* has also reported a high liver uptake of ^225^Ac in mice and rats [43].

Radium naturally targets the hydroxyapatite matrix in the bone and is thus the ideal agent for the treatment of bone metastasis. The rapid cascade of alpha particle emitting daughters will thus deliver high doses to bone tumours. Although the daughter nuclides are not intrinsically bone-seeking, their short half-life appears to prevent them from doing major damage to healthy tissue. An *in vivo* study has shown that less than 2% of the daughters migrate away from the bone surface within 6 h after administration of ^223^Ra, and after three days this number has dropped down to less than 1% [24]. This effect is due to the long half-life of ^223^Ra, such that after a few days it has been incorporated better into the bone tissue. The retention of the daughters in bone thus appears similar to that of the parent nuclide. However, when ^223^Ra would be targeted to other sites than bone tumours, recoiling daughters would be more likely to escape from the targeted site and distribute according to their biological affinity, once again depending on half-life and location.

To learn more about the effects observed in the group of AS patients treated with ^224^Ra as described earlier, Lloyd *et al.* have attempted to acquire more information about the biodistribution of ^224^Ra and its daughters [44]. In their study, six young adult beagles were killed from 1 h to 7 days after a 3.4 MBq ^224^Ra injection, and activities of ^224^Ra, ^212^Pb, and ^212^Bi were counted in humerus, lumbular vertebrae, eyes, kidneys, and the liver. Biological retention of the ^224^Ra in the bones ranges from 37% at 1 h till 58% after 8 h, decreasing slowly to 42% after a week. In the soft tissue on the other hand, 61% of the initial activity has been found 1 h after administration, diminishing to only 6% at 7 days. About 8% of the ^220^Rn gas has left the body. Furthermore, substantial amounts of ^212^Bi and ^212^Pb have been found in red blood cells. The kidneys contained mainly ^212^Bi, and the liver had excess amounts of ^212^Pb (20% of total) as compared to ^224^Ra. The eyes and bones contained relatively less ^212^Bi and ^212^Pb than ^224^Ra. These results indicate significant redistribution of the daughter nuclides. This higher redistribution as compared to that found in ^223^Ra (t_1/2_ = 11.4 days) studies can be partially explained by the shorter half-life of ^224^Ra (t_1/2_ = 3.7 days), as the mother isotope does not have enough time to move deep into the bone surface for the daughters to be also retained in the bone. In addition, the gaseous daughter ^220^Rn (t_1/2_ = 55 s) has a much longer time to redistribute itself in the body as compared to ^219^Rn (t_1/2_ = 3.9 s), the ^223^Ra daughter. These are the two main reasons that ^224^Ra therapy has shown adverse effects in the kidneys, eyes and livers of treated patients [44].

## 4. Approaches to Deal with the Recoil Problem

There are generally speaking three strategies proposed in literature to deal with recoil issues: encapsulation in a nano-carrier, fast uptake of the alpha emitters in tumour cells, and local administration such as intratumoural injection.

### 4.1. Encapsulation in A Nano-Carrier

The first approach is to apply some kind of a nano-carrier that is capable of retaining the recoiling daughters. So far zeolites, liposomes, polymersomes, and metal-based particles have been investigated. The actual recoil retention is difficult to be compared though, as determining the percentage of recoiling daughters is not always straightforward, especially in systems where re-association is possible. For instance, Piotrowska *et al.* have used zeolites as carriers for ^224^Ra, showing that the percentage of recoiled daughters (^212^Bi, ^212^Pb and ^208^Tl) escaping from the zeolites is relatively small even under blood serum conditions [45]. However, it needs to be mentioned that in these experiments, most likely, equilibrium has been established and the determined values in fact reflect the distribution of the different ions according to their stability constants. Once injected in the blood stream, the ejected radionuclides will not be in thermodynamic equilibrium anymore and their distribution might be of a very different kind.

Sofou *et al.* have used different types of liposomes (zwitterionic, cationic and anionic) to perform retention studies of ^225^Ac and its daughters. ^213^Bi retention has been revealed to have weak size dependence for liposomes sized between 100 and 800 nm a maximum of 12% remained in the carriers [46]. At the same time, Wang *et al.*, have investigated polymeric vesicles for the same decay chain and have reported that nearly 70% of ^213^Bi is still present in carriers of 800 nm [47]. These discrepancies can be due to the relatively fast decay of ^225^Ac that creates experimental difficulties prone to measurement errors strongly dependent on the separation method. In the case of ^225^Ac decay, the separation method should be fast enough otherwise the initial activity of ^221^Fr and ^213^Bi might be wrongly calculated, resulting in underestimation of the recoil retention. Typically after separation the time is monitored accurately and the activity of ^221^Fr and ^213^Bi is determined and plotted against time. The initial activity is then estimated using a simplified version of the secular equilibrium decay equation: $A_{2}\left(t\right)=\frac{{\text{λ}}_{2}}{{\text{λ}}_{2}-{\text{λ}}_{1}}A_{1}\left(0\right)\left(e^{-{\text{λ}}_{1}t}-e^{-{\text{λ}}_{2}t}\right)+A_{2}\left(0\right)e^{-{\text{λ}}_{2}t}$, where *A_1_* and *A_2_* reflect the activity of the mother and daughter respectively, while λ_1_ and λ_2_ correspond to the mother and daughter decay constants. A slow separation does not allow for determining the ^221^Fr escape and leads to uncertainties of the initial ^213^Bi concentration, *i.e.*, the nearer the measured time is to time zero the more accurate the determination of the initial activity and hence the retention.

Liposomes of 80 nm in size have also been investigated as carriers of ^223^Ra, but unfortunately all available recoil data is based on *in vivo* experiments, which complicates determining the actual retention. Nevertheless, from this study is clear that that the recoil daughters ^211^Pb and ^211^Bi (the third and fourth daughter of ^223^Ra) are not retained sufficiently in the liposomes, as the activity measured in the kidneys has been found to be much higher than the expected value based on equilibrium assumptions, calculated from the ^223^Ra activity in the same organ. In addition to this ^223^Ra has also been measured in the bones of the lab-animals. These findings strongly suggest that relatively small sizes of this type of soft nano-carriers will not retain a sufficient percentage of recoils, which can also be anticipated from range calculation of recoiling atoms based on their stopping power [48]. In soft materials like polymers, for instance, the projected range of ^221^Fr has been reported to be around 90 nm, which can already be larger than the applied nano-carrier [37]. An alpha cascade will result in an even larger distance that the recoils can cover. Therefore in order to achieve sufficient recoil retention materials having higher Z are necessary. This line of reasoning has been followed by Woodward *et al.* and McLaughlin *et al.*, both working on lanthanide-based phosphate nanoparticles doped with ^225^Ac, except the latter has also applied gold coating to the carriers [49,50]. The retention of ^221^Fr has been determined *in vitro*, while the fate of ^213^Bi has also been assessed *in vivo*. The gold-layered particles have given the most promising results with nearly 100% initial retention of ^221^Fr when four layers of gadolinium phosphate are applied, but unfortunately these particles could not be used *in vivo* due to sedimentation issues. Biodistribution studies based on ^225^Ac-nanoparticles having less than four gadolinium phosphate layers coupled to mAb 201b have revealed a ^213^Bi retention of at least 69% and a much-decreased renal uptake of free ^213^Bi. Although these results are very promising, there is still liver uptake of the ^225^Ac doped particles due to their size. Considering the nature of the particles it is expected that they will remain in the liver for a considerable time, which might cause adverse effects and should be studied further. Smaller particles may prove to have better bio-distribution and faster clearance but on the expense of recoil retention. Clearly an optimum between recoil retention and uptake of nano-carriers by healthy organs needs to be determined to make this approach applicable in the clinic.

### 4.2. Fast Uptake in Tumour Cells

The second approach is to ensure that the radiopharmaceutical is rapidly taken up by the tumour cells and that the remaining not-adsorbed part is excreted fast from the body. This argument has been used for both peptides and antibodies. Since antibodies take a long time to accumulate at the tumour site, one can imagine that smaller targeting agents would fit better to this strategy. Nevertheless, this approach is almost entirely applied with larger targeting agents. Similarly in the case of ^223^Ra and its daughters, their affinity to bone can be used to limit the toxicity to other tissues.

^225^Ac has been investigated in conjugation with vascular tumour-homing peptide F3 [51] and with octreotide [52] in mice resulting in a rather different kidney toxicity. In the case of the F3 peptide little differences have been observed between ^225^Ac and ^213^Bi concerning both therapeutic efficiency and kidney toxicity. The octreotide results have revealed better efficiencies when compared to ^177^Lu in the treatment of neuroendocrine tumours, but tubular necrosis at activities above 30 kBq has been reported. The biodistribution in this case has shown high tumour uptake within 30 minutes and absence of blood activity within 4 h after injection. The kidney uptake, although rapidly decaying since the injection time point, has been found to be still above 5%ID/g at 24 h [52]. No biodistribution data has been provided in the F3 peptide study. An explanation for these toxicity differences can be the rather larger activities of ^225^Ac applied with octreotide, *i.e.*, 30 kBq *vs.* 1.85 kBq used in the F3 peptide conjugation. In addition to this the mice in the F3 peptide studies have been followed only for 3 months unlike in the octreotide experiments where a period of 7 months has been chosen, which implies that any retarded effects could simply not have been detected in the first case.

Significantly more attention has been paid to the use of antibodies in alpha radionuclide therapy. One of the first pre-clinical studies using ^225^Ac has been carried out by McDevitt *et al.* who aimed at a high degree of tumour cell internalization as a solution to the recoil problem [53]. *In vivo* experiments have been performed using two different animal models, prostate carcinoma and disseminated lymphoma, and respectively antibodies J591 and B4 conjugated to DOTA. In addition to this in the first model the actual retention of ^221^Fr and ^213^Bi has been estimated in the tumour tissue, revealing the presence of 88% of ^221^Fr and 89% of ^213^Bi of the ^225^Ac secular equilibrium values. The results of these studies indicated a clear benefit of alpha radiation at low administered activities with no toxic effects. Further in this investigation the *in vitro* stability of Ac-DOTA bound to lintuzumab (HuM195) has been determined revealing little dissociation of ^225^Ac. The same antibody coupled to ^225^Ac has also been applied in experiments concerning non-human primates. The half-life blood circulation time of the antibody has been found to be 12 days with 45% blood clearance of ^213^Bi. In addition to this at low activities (28 kBq/kg) no toxicity has been observed within 6 months, but at cumulative doses of 215 to 370 kBq severe toxic effects become apparent. These toxic effects are believed to be caused by the released daughters from the unbound antibody, ^221^Fr and ^213^Bi, which are expected to accumulate in the kidneys [54]. These results suggest that remaining below activities of 28 kBq/kg should not lead to adverse effects and the combination of this antibody with ^225^Ac has actually been translated to clinical studies as described earlier in this paper [55].

^225^Ac in combination with trastuzumab, an anti-HER2/neu antibody, has also been tested in ovarian cancer mice models applying intraperitoneal injection and three levels of activities: 8.1, 12.2 and 16.7 kBq. The results suggest that fractionated therapy using less activity might be more advantageous than a single injection of the same total activity based on early mortality numbers and median survival time, which appeared to be optimal when administering less activity. The treatment has been concluded to be successful because of the reached significant extension of the survival time and limited toxicity [56].

^225^Ac coupled to anti rat HER-2/neu monoclonal antibody (7.16.4) has been studied in the treatment of breast cancer metastases. The therapeutic results have shown remarkable prolongation of mice survival time, *i.e.*, up to 1 year, and better efficiency than ^213^Bi or ^90^Y controls. However, the biodistribution findings have indicated serious damage of the kidneys, probably caused by the two daughters of the alpha emitter having the longest half-life, *i.e.*, ^221^Fr and ^213^Bi [15].

The success of therapy with ^225^Ac will depend on the extend to which renal toxicity can be reduced. In principle, renal damage can be greatly diminished by means of administering a metal scavenger and a diuretic as reported by Jaggi *et al.* [38]. In this case the metal chelate (2,3-dimercapto-1-propanesulfonic acid, DMPS) administered orally has significantly suppressed ^213^Bi kidney uptake by, most likely, complexing with the metal and prolonging its circulation time in the blood. On the other hand, diuretics such as furosemide and chlorothiazide have been used to inhibit the absorption of ^221^Fr in renal tubular cells showing that excretion is stimulated and kidney damage is reduced.

Besides ^225^Ac, ^227^Th conjugated to the monoclonal antibody DAB-4 has been evaluated in combination with chemotherapy in mice with induced Lewis lung tumours. Mice have had better median survival times even when ^227^Th has been applied on its own with activities of 20 kBq, but the therapeutic efficiency has been shown to be more pronounced when combined with chemotherapeutics. Adverse health effects have not been observed but considering the very short survival times of the mice, (*i.e.*, in the best case 19 days), this is not surprising and the authors have acknowledged that bone uptake might become a problem in malignant cases having longer life expectancy [57]. Indeed the development of secondary cancers might be expected considering the fact that ^223^Ra is the daughter of ^227^Th and that it accumulates in bone but no data could be found in this particular publication to support these expectations. Further elaborate studies concerning the toxicity of ^227^Th coupled to rituximab have been carried out by Dahle *et al.* who investigated different activity levels and their effect on mouse body weight, white blood cells and platelet counts, and histological examination. The no-observed-adverse-effect-level has been found to be 200 kBq/kg, although the maximum tolerated activity has been estimated to be much higher, *i.e.*, between 600 and 1000 kBq/kg. The contribution of ^223^Ra to bone marrow toxicity could not be properly evaluated due to cross-contaminations [33].

### 4.3. Local Administration

The third approach is to insert or inject the alpha-emitting radionuclides directly in the or near the tumour tissue, as has been tested in Phase I clinical studies with ^213^Bi-DOTA-substance P locally injected in gliomas by Cordier *et al.* [7]. This approach is also used by Cooks et al, who have made clever use of the recoiling properties of alpha emitting radionuclides in a series of studies with ^224^Ra [58,59,60,61,62]. They have developed ^224^Ra-loaded wires that continuously release radioactive daughters ^220^Rn, ^216^Po and ^212^Pb from their surface, while the ^224^Ra itself remains in the wires. According to their theoretical studies, the region around the site of wire insertion affected by the diffusion of ^220^Rn, the recoiled daughter of ^224^Ra, is in the order of millimetres, reaching a dose of 10 Gy up to 2.6 mm away from the inserted wire at an initial ^220^Ra activity of 37 kBq. Experimental studies show that the ^220^Rn daughter ^212^Pb travels substantially further away from the tumour. In 0.1 g tumours, up to about 90% of the ^212^Pb leaks from the tumours, while for 2.4 g tumours this is only 12%. Most of the ^212^Pb leaving the tumour is absorbed by the kidneys (8.5 ± 5.8%) [58]. Despite the high kidney dose, this new form of “brachytherapy”, called diffusing alpha-emitters radiation therapy (DaRT), has shown great promise in the treatment of both pancreatic [61], and lung carcinomas [59], as well as a number of other human-derived tumours [59,60]. Recently, an anti-tumour immunity in mice previously treated with DaRT therapy has been found, where 77% of the population did not develop tumours after re-inoculation of the tumour cells, 21 days after the treatment with the ^224^Ra wires (versus 33% in the control group). The survival of DaRT-treated mice has increased from 29% (untreated) to 63% [62], suggesting that improved long-term cancer immunity might arise from tumour antigens supplied by destroyed tumour cells. This form of therapy could prove very beneficial for patients with relatively large, accessible tumours, though for smaller metastasis different methods would need to be applied.

## 5. Conclusions

This paper has presented a review on alpha radionuclide therapy. During the past few years, some progress has been made in getting a few alpha radionuclide therapy concepts to the clinic, however, by and large we find that in most studies concerning long-lived alpha-emitters, recoiling daughter atoms pose a serious problem. If the distribution of recoiled atoms is not controlled, toxic effects due to the recoiling daughters will be present. At higher injected radionuclide activities, this could induce significant damage to healthy tissue. The question is whether the activity that can be injected without toxic effects will be sufficient to have the expected (much improved) therapeutic effects. The success of each approach depends to a large extent on the type of radionuclide (e.g., ^223^Ra) and the type of tumours to be treated. For instance, in systemic radionuclide therapy to treat metastasized tumours other than bone malignancies, nano-carriers that can limit the distribution of the recoiling daughters may offer a solution. In this case, the carriers that do not accumulate at the tumour site should also not be retained in healthy organs. Rapid clearance of these particles from the organs is thus essential. On the other hand, tumours that are easily accessible can profit from direct insertion of the alpha-emitter in a tumour volume. Alpha-radionuclide therapy is thus promising, provided that the recoil obstacle can be overcome first. Only then will patients have the full benefit of this treatment.
